# Amplified Fragment Length Polymorphism Mapping of Quantitative Trait Loci for Economically Important Traits in the Silkworm, *Bombyx mori*

**DOI:** 10.1673/031.010.14113

**Published:** 2010-09-14

**Authors:** Seyed Z Mirhoseini, Babak Rabiei, Payam Potki, Seyed B Dalirsefat

**Affiliations:** ^1^ Department of Animal Science, Faculty of Agricultural Sciences, University of Guilan, PO Box 41635-13 14, Rasht, Guilan, Iran; ^2^ Department of Agronomy & Plant Breeding, Faculty of Agricultural Sciences, University of Guilan, PO Box 41635-1314, Rasht, Guilan, Iran; ^3^ Department of Genomics, Agricultural Biotechnology Research Institute of Iran (Rasht), PO Box 41635-41 15, Rasht, Guilan, Iran; ^4^ Department of Sericulture, Faculty of Agricultural Sciences, University of Guilan, PO Box 41635-13 14, Rasht, Guilan, Iran

**Keywords:** AFLP markers, cocoon traits, QTL mapping

## Abstract

Cocoon related characteristics are economically important traits in the silkworm, *Bombyx mori* L. (Lepidoptera: Bombycidae). In this study a genetic linkage map was developed that identified QTL controlling the cocoon weight, cocoon shell weight, and cocoon shell percentage using 161 amplified fragment length polymorphism (AFLP) markers. Twenty *Pst*I/*Taq*I primer combinations were employed to genotype 78 F_2_ progenies derived from a cross between P107 Japanese inbred line and Khorasan Lemon Iranian native strain. Among polymorphic markers, 159 AFLP markers were assigned to 24 linkage groups at the LOD threshold of 2.5 that varied in length from 4 to 299 cM. The total length of the linkage map was 2747 cM, giving an average marker resolution of 19.31 cM. A total of 21 AFLP markers were identified that were distributed over the ten linkage groups linked to the three studied traits using the composite interval mapping method. The explained variation rate by QTL controlling cocoon weight, cocoon shell weight, and cocoon shell percentage ranged from 0.02% to 64.85%, 0.2% to 49.11%, and 0.04% to 84.20%, respectively. These QTL controlled by different actions as well as under dominance, additive, partial dominance, dominance, and over dominance.

## Introduction

The silkworm, *Bombyx mori* L. (Lepidoptera: Bombycidae), domesticated for silk production for about 5000 years, is an agriculturally important insect and comprises a large number of geographical races and inbred lines that show substantial variation in their qualitative and quantitative traits (Mirhoseini et al. 2007). With the establishment of stable transformation (Yamao et al. 1999; Tamura et al. 2000), *B. mori* has shown the potential to produce pharmaceutically important proteins in high yield (Tomita et al. 2003), opening up new applications for sericulture in medical, agricultural, and industrial fields (Yamamoto et al. 2006). Currently, it is the major economic resource for nearly 30 million families in countries such as China, India, Vietnam, and Thailand (Miao et al. 2005). In order to make sericulture economically viable, genes affecting growth rate, yield, fiber quality, and virus resistance can be tagged with molecular markers for rapid construction of genetically improved strains. Taking the exclusive investigational advantages of this organism into account, as well as its economic importance, an International Consortium on Lepidopteran Genomics was formed a few years ago to support international cooperation to sequence the genome of *B. mori* and to initiate comparative genomics of other economically important Lepidoptera (Nagaraju and Goldsmith 2002).

Most traits in nature and of importance to agriculture are quantitatively inherited and therefore are difficult to study due to the complex nature of their inheritance. However, recent advances of genomic technologies have led to revolutionary means for unraveling the secrets of genetic variation in quantitative traits. Genomic technologies allow the molecular characterization of polymorphic markers throughout the entire genome that are then used to identify and map the genes or quantitative trait loci (QTL) underlying a quantitative trait based on linkage analysis (Wu et al. 2007).

A complete linkage map is necessary to efficiently carry out molecular-based analyses such as molecular marker-assisted selection, quantitative trait loci (QTL) mapping of agronomically important traits, prediction of heterosis, and comprehensive investigations of genomic evolution between lineages (Tan et al. 2001). Presently, genome studies in *B. mori* have generated genetic linkage maps based on morphological markers (Doira et al. 1992) and molecular markers including RFLP (Goldsmith 1991; Shi et al. 1995; Nguu et al. 2005), RAPD (Promboon et al. 1995; Yasukochi 1998; Li et al. 2000), SADF and RAPD (He et al. 2001), AFLP (Tan et al. 2001; Lu et al. 2004; Sima et al. 2006), microsatellites (Miao et al. 2005), and SNP (Yamamoto et al. 2006).

Projects have been initiated to find molecular markers that are tightly linked to traits relevant for sericulture, with the related goals of developing tools for marker assisted selection and positional cloning. RAPD or cDNA markers have been associated with the four known densonucleovirus nonsusceptibility loci, *nsd-1* (Abe et al. 1998), *nsd-2* (Abe et al. 2000), *Nid-1* (Kadono-Okuda et al. 2003), and *nsd-Z* (Li et al. 2001). Two large contigs on chromosome 17 that encompass cDNAs closely linked to *Nid-1* and *nsd-2* have been isolated and sequenced (Kadono-Okuda et al. 2003) and are being examined for candidate genes in susceptible and nonsusceptible strains (Goldsmith et al. 2005). A similar strategy was used to screen for RAPD markers linked to resistance to NPV, a potentially devastating pathogen (Yao et al. 2003), and fluoride resistance (Chen et al. 2003). Progress has also been made in assigning RAPDs (Chatterjee and Pradeep 2003), inter-simple sequence repeat markers (Chatterjee and Mohandas 2003), and AFLP (Lu et al. 2004; Li et al. 2006; Sima et al. 2006) to QTL for characters such as larval growth rate and pupal and cocoon weight. A suite of additional fingerprinting tools has been developed for these applications (Nagaraju and Goldsmith 2002). In addition, a collection of about 8500 expressed sequence tags (ESTs) is now available in GenBank and provides an additional source of important anchors in the ongoing *Bombyx* genome study (Nguu et al. 2005).

Although for genome mapping, the ideal genetic marker is codominant, multiallelic, and hypervariable (i.e., segregates in almost every family), some dominant markers are also very useful and powerful in particular situations (Wu et al. 2007). The amplified fragment length polymerphism (AFLP) technique (Zabeau and Vos 1992; Vos et al. 1995) has demonstrated to be a convenient and reliable tool to generate highly polymorphic molecular markers that greatly facilitate building linkage maps (Qi et al. 1997; Waugh et al. 1997). AFLP markers do allow one to construct linkage maps with wide genome coverage without engaging in extensive sequencing or marker development programs. AFLP markers are also faster than individual codominant marker types because a single polymerase chain reaction (PCR) can derive multiple loci simultaneously (Erickson et al. 2004). Because of these features, AFLP has been widely employed for genetic mapping in various organisms.

In this study, significant molecular markers and a large segregating population size were employed to detect QTL linked to economically important traits relevant to the *B. mori* cocoon and to better identify the genome regions of these QTL. Since high-resolution QTL mapping is critical for positional cloning and gene isolation (Zhong et al. 2006), a high resolution AFLP-based genetic linkage map and the results of QTL mapping for economically important cocoon traits are reported.

## Materials and Methods

### Insect materials and crosses

One F2 segregating family from mating between a Japanese inbred line (P107) as female parent and an Iranian native strain (Khorasan Lemon) as male parent were used in the study. These inbred line and strain exhibit high phenotype diversity for economically important characters such as whole cocoon weight, cocoon shell weight, and cocoon shell percentage, suggesting that considerable polymorphism exists at the DNA level (Dalirsefat and Mirhoseini, 2007). Indeed, the highest and the least quantities of these traits corresponded to P107 and Khorasan Lemon, respectively. These inbred line and strain have undergone a high degree of inbreeding and are relatively homozygous. A total number of 78 progenies, including 39 males and 39 females from F_2_ population, were used to construct the genetic linkage map and QTL detection. The parents and F1 progenies were used to establish the segregation pattern of the molecular markers. The crossing experiments were done in the Iran Silkworm Research Center (ISRC) located in Rasht, Iran.

### AFLP analysis

Genomic DNAs were isolated individually from all the parents, F1, and F_2_ populations, in the moth stage following the phenol/chloroform method (Suzuki et al. 1972) and as modified by Nagaraja and Nagaraju (1995). DNAs were quantified using a known standard (DNA lambda, Roche, www.roche.com) on agarose gels.

All individuals were subjected to genotyping with AFLP markers according to Vos et al. (1995) with some modifications. Briefly, genomic DNA was double digested with *Pst*I and *Taq*I restriction enzymes, which produce polymorphic DNA fragments in *B. mori* (Tan et al. 2001; Mirhoseini et al. 2007). The DNA fragments were ligated with *Pst*I and *Taq*I adaptors, generating template DNA for PCR amplification. Two primers were designed on the basis of adaptor sequences and restriction site sequences to use in PCR amplification. Selective nucleotide sequences were added to the 3′ end of each primer. PCR amplification was conducted in two steps: a pre-amplification and a selective amplification. For the selective amplification, a total of 81 primer combinations obtained from two sets of *Pst*I and *Taq*I selective primers (Table 1) were screened. Among them, 20 primer pairs that produced fragments with clear dominance inheritance patterns and reproducibility were used for the linkage analysis. Polymorphism screening of AFLP products was conducted on a 6% Polyacrylamide gel using SequiGen 38×30 cm gel apparatus (BioRad Laboratories Inc., www.bio-rad.com). Bands were detected by the silver staining procedure (Promega, www.promega.com, Technical manual No.023), and gel images were scanned and saved as jpeg files for scoring and further analysis.

Linkage analysis and map construction Using genotype information of 81 AFLP primer combinations, 20 primer combinations that produced clearly readable and polymorphic fragments among parents were employed to analyze linkage mapping. Twenty polymorphic primer combinations generating 161 polymorphic AFLP fragments with a clear dominance inheritance pattern were employed to construct the linkage map and detect QTL; that is, the suitable fragments must show complete dominance expression in one parent and complete recessive expression in the other, and all F1 individuals must be heterozygous. The AFLP fragments were scored based on 0 and 1 and then converted to A, B, C, and D letters according to the Map manager QTX (Manly et al. 2001) instruction manual. The data were analyzed using the Kosambi map function (Kosambi 1944) of Map manager QTX (Manly et al. 2001) to develop a linkage map for the population. By genotyping 78 progenies from the F2 population using 161 polymorphic bands, a genotypic data matrix in a dimension of 78 × 161 was constructed and used for linkage mapping. First recombination rates among markers were evaluated, and then recombination rates converted to the map distance based on centiMorgan using the Kosambi map function (Kosambi 1944). Computer software QTL cartographer version 2.5 (Wang et al. 2007) was used to determine the QTL positions, the expected additive and dominance effects, and the phenotypic variance explained by individual QTL. The LOD threshold value for declaring the presence of a QTL was determined by a permutation test (*n* = 1000) (Churchill and Doerge 1994). Genome-wide threshold levels were used to declare significant QTL based at the 5% significance level. Average levels of dominance (h) were estimated using the ratio dominance/additive effects (Stuber et al. 1987).

All molecular experiments were established in the genomics laboratory of the Agricultural Biotechnology Research Institute of North Region (Rasht) under the supervision of the Agricultural Biotechnology Research Institute of Iran (ABRII).

## Results

### Linkage map construction

**Figure 1.**
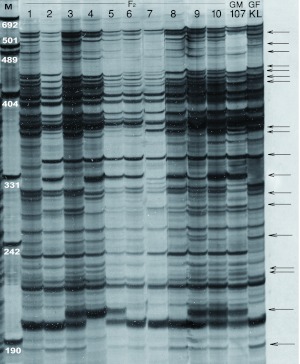
An example of AFLP gel electrophoresis and polymorphism screening corresponding to the Ptat- Ttac primer combination. GM-107 and GF-KL represent female and male parents, respectively. M stands for standard molecular size marker. Polymorphic bands are shown by arrows. High quality figures are available online.

Among the 81 AFLP primer combinations screened, approximately one-third of the primer combinations (*n* = 28) produced polymorphic fragments between the P107 inbred line and the Khorasan Lemon native strain. Twenty pairs of AFLP primer combinations were selected for segregation analysis on the F2 population based on reproducibility and the degree of polymorphism. Only the polymorphic fragments that segregated in a dominant manner and could be scored unambiguously were used for linkage map construction. An example of AFLP gel electrophoresis and polymorphism screening related to the Ptat-Ttac primer combination is shown in Figure 1.

**Table 1.**
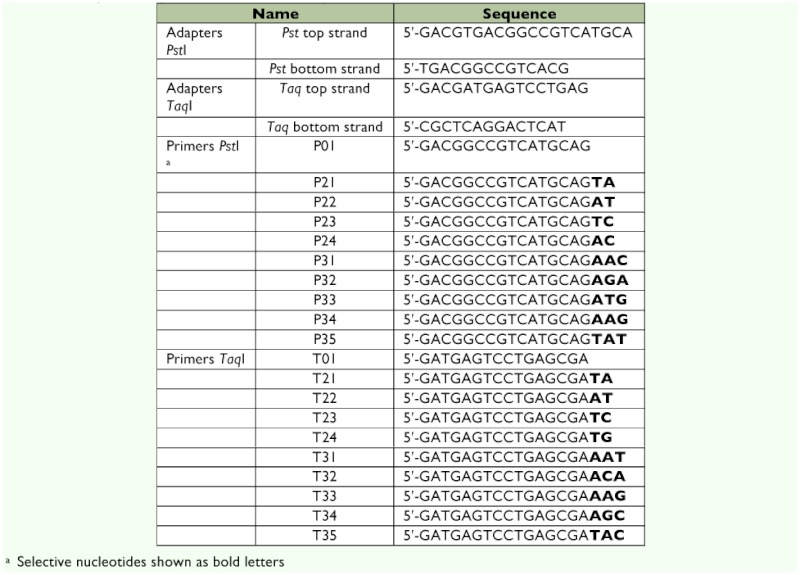
Adapters and primers used in AFLP analysis.

Twenty *Pst*I/*Taq*I primer combinations produced 845 clearly detected bands, of which 161 qualified polymorphic fragments showing good agreement of 3:1 segregation (for a dominant marker, the segregation ratio is 3:1 in the F2 population) were analyzed for linkage mapping. The frequency of polymorphic AFLP markers derived from the clearly detected bands in the P107 × Khorasan Lemon cross in the silkworm was 19.35%. This frequency was close to that obtained in the Dazao × C100 cross of the silkworm (25.7%) (Lu et al. 2004) but it was dramatically lower than in the no. 782 × od100 cross (60.7%) of the silkworm (Tan et al. 2001).

91 fragments of 161 polymorphic fragments (56.52%) were detected in the male parent (Khorasan Lemon strain), and 70 fragments (43.48%) were observed in the female parent (P107 inbred line). On average, each primer combination generated 8.05 polymorphic fragments that could be used for linkage mapping. The number of polymorphic bands produced using the 20 primer combinations ranged from 3 bands (7.32%) corresponding to P33-T32 to 14 bands (23.33%) for P22-T31 (Table 2).

**Figure 2.**
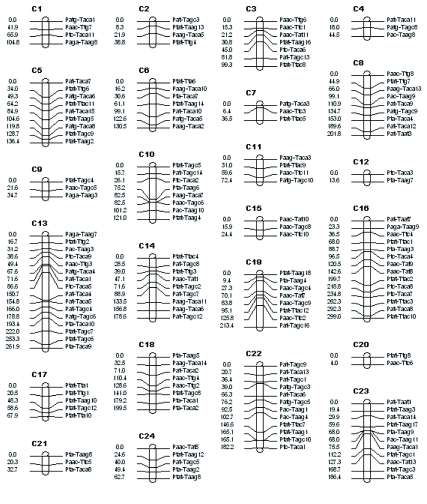
AFLP Linkage map of the silkworm (Bombyx *mori*) based on 76 F2 males derived from the cross between P107 and Khorasan Lemon. The numbers on the left side of each linkage group are genetic distances in Kosambi centiMorgans. AFLP markers are designated by the *Pst*I and *Taq*I primer names on the right side of each linkage group. High quality figures are available online.

The linkage map generated from the P107 × Khorasan Lemon cross contained 159 AFLP markers (two markers were unlinked) that were assigned to 24 linkage groups at the LOD threshold of 2.5 (Figure 2). Average distance between markers was 19.31 cM. The total recombination distance over 24 linkage groups was 2747 cM, which was longer than previous estimates in *B. mori* i.e. 1800 cM for the dense RAPD map (Yasukochi 1998), 1868.10 cM and 2677.50 M for the AFLP maps in two F_2_ subgroups (Sima et al. 2006), and 1305 cM for SNPs based linkage map (Yamamoto et al. 2006). However, it was shorter than 6512 cM (Tan et al. 2001) and 3676.7 cM (Li et al. 2006) for the AFLP maps and 3431.9 cM (Miao et al. 2005) for the SSR markers reported in backcrossed populations of *B. mori.* Miao et al. (2005) suggested that although many conditions influence map length, including differences in mating strategy and strains used, the distribution of markers is a possible causative aspect, and increased marker density should converge on a more realistic map length value. As Tan and Ma (1998) demonstrated theoretically, with additional markers typed, the map length may increase when marker density is not saturated or may decrease when marker density is in a saturation state (Tan et al. 2001). For example, Causse et al. (1994) constructed a rice map with 762 markers covering 4026.3 cM, whereas Harushima et al. (1998) obtained a 2275-marker genetic map of rice covering 1521.6 cM. This may explain why the length of our AFLP map is more than that of the *B. mori* linkage map studies mentioned above except for the maps of Tan et al. (2001), Miao et al. 2005, and Li et al. (2006).

Considering that the estimated genome size of *B. mori* is 530 Mbp (Gage 1974), the average physical distance per recombination distance is about 193 kb/cM. It seems that the AFLP markers did not exhibit significant clustering near centromeres or the distal region of chromosomes, suggesting that they provide good coverage of the genome (!”#$% et al. 2006, Figure 2).

**Table 2.**
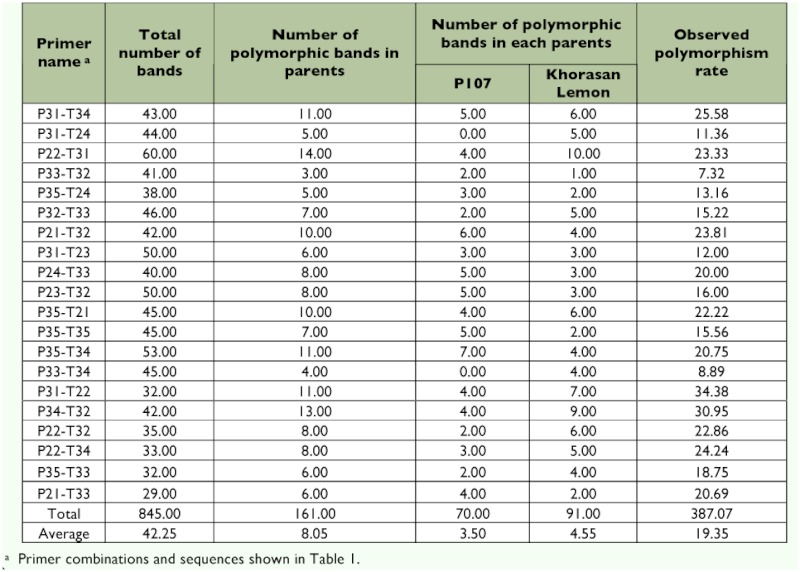
The observed polymorphisms of twenty *Taq*I and PstI primer combinations used in the parents and F2 population.

### Phenotypic values

The average phenotypic values of cocoon weight, cocoon shell weight, and cocoon shell percentage traits corresponding to each parent and F_2_ offspring are shown in Table 3. An extremely high significant difference (p < 0.01) for these traits was revealed as a result of comparing the mean phenotypic values between parents using the *t*-test. The mean cocoon weight in line P107 as female parent and Khorasan Lemon as male parent was 1.479 and 1.404 g, respectively. The mean cocoon shell weight in line P107 was 0.324 g, approximately 0.113 g more than that in Khorasan Lemon (0.211 g). In addition, the cocoon shell percentage in the line P107 was estimated to be 22.23%, which was seven percent more than that in Khorasan Lemon (15.23%) as the male parent. Except for cocoon weight value in the F_2_ population, which was higher than both parent values, both the mean cocoon shell weight and cocoon shell percentage traits in the F_2_ generation were closely equal to the mean parent values (Table 3).

**Figure 3.**
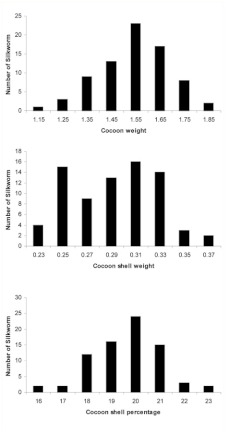
Frequency distribution of phenotypic values related to the studied traits in the F2 segregating *Bombyx mori* population derived from mating between the P107 inbred line and the Khorasan Lemon native strain (the mean phenotypic values of the parents for the three traits are shown in Table 3). High quality figures are available online.

The frequency distribution of phenotypic values related to each trait studied in the F_2_ offspring is illustrated in Figure 3. As it is shown, a number of F_2_ offspring demonstrated out of range parent values, especially in the line P107 for the three studied traits. The least and the highest phenotypic values of cocoon weight in the F_2_ population were 1.13 g and 1.83 g, respectively, and the highest value was about 0.35 g more than that in the line P107 (1.479 g). The least and the highest phenotypic values of cocoon shell weight in the F_2_ population were 0.22 g and 0.37 g, respectively; consequently, a number of the F_2_ offspring had almost 0.046 g more than that in the line P107 phenotypic value (0.324 g). In addition, the least and the highest phenotypic values of cocoon shell percentage in the F_2_ progenies were 15.72% and 22.6%, respectively; the highest value was nearly 0.4% higher than that in the line P107 (22.2%) (Figure 3).

### QTL analysis

A total of 21 different loci, including QTL, controlling cocoon weight, cocoon shell weight, and cocoon shell percentage traits were detected in the linkage map using a composite interval mapping method at the LOD threshold of 2.5 (Table 4). The selected LOD score plots at the threshold of 3 for linkage groups with the identified QTL provided a basis for identifying the molecular markers most closely linked to the QTL (Figure 4).

In particular, 12 QTL controlling cocoon weight were identified on the LG1, LG5, LG6, LG8, LG16, LG17, and LG19. The additive effects of these QTL ranged from -0.1581 (*cw8*) to +0.0887 (*cw19b*), and their dominance effects ranged from -0.3852 (*cw19a*) to +0.3881 (*cw1a*). Two QTL for cocoon shell weight were identified and located on the LG16 and LG22 with additive effects ranging from -0.0164 (*cshw16c*) to +0.0459 (*cshw22b*) and dominance effects ranging from -0.0314 (*cshw16c*) to +0.037 (*cshw22b*). Finally, fourteen QTL were identified for cocoon shell percentage located on the LG8, LG9, LG16, LG19, and LG23. The additive effects of these QTL ranged from -0.0347 (*cshp23b*) to +0.0118 (*cshp8*), and their dominance effects ranged from -0.0361 (*cshp8*) to + 0.0352 (*cshp19a*) (Table 4).

**Table 3.**
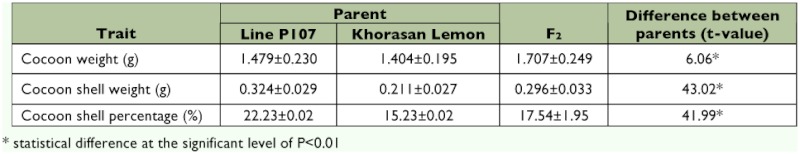
Mean phenotypic values of traits studied in the parents and F2 population.

**Table 4.**
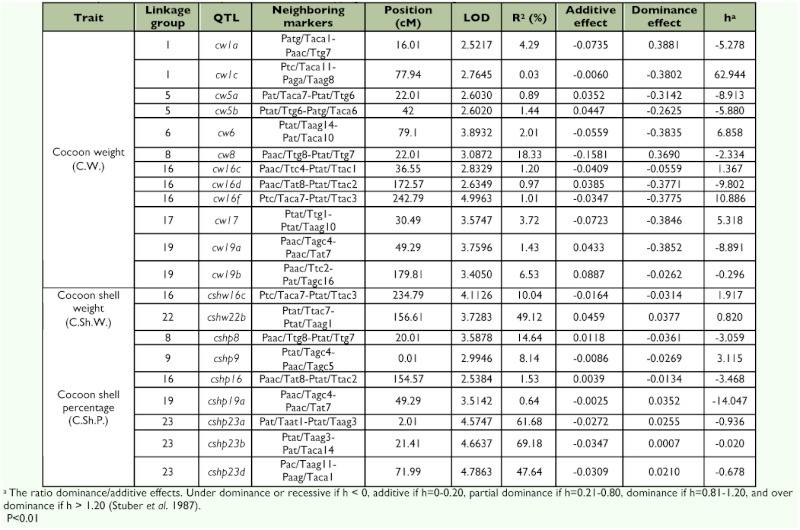
Genetic parameters as estimated by composite interval mapping of the QTL affecting the cocoon related traits in the silkworm.

The explained variation rate by QTL controlling cocoon weight, cocoon shell weight, and cocoon shell percentage ranged from 0.02% to 64.85%, 0.2% to 49.11%, and 0.04% to 84.20%, respectively (Table 4).

## Discussion

In the present study, an AFLP-based linkage map containing 159 AFLP markers in a total length of 2747 cM and an average marker resolution of 19.31 cM was developed for *B. mori.* Using this map, a total of 21 AFLP markers linked to cocoon weight, cocoon shell weight, and cocoon shell percentage were identified using a composite interval mapping method (Table 4). Recently, 11 QTL (Lu et al. 2004) and 40 QTL (Li et al. 2006) for whole cocoon weight, cocoon shell weight, ratio of shell weight and weight of pupae have been reported, and Javadi Taklimi (2006) accounted 5 QTL controlling ratios of shell weight in a backcrossed population (BC1) of *B. mori.* These differences may be due to sample size, number and type of primer combinations used, and crosses established. Though apart from employing different primer pairs, Lu et al. (2004) and Li et al. (2006) used 44 BC1 progenies, and Javadi Taklimi (2006) applied only seven polymorphic primer combinations. Practically, factors such as the number of molecular markers used, types of crosses, sample size of segregating population, number of genes controlling the traits, and existence of gene interaction may influence the statistical power of QTL mapping (Zhong et al. 2006).

**Figure 4.**
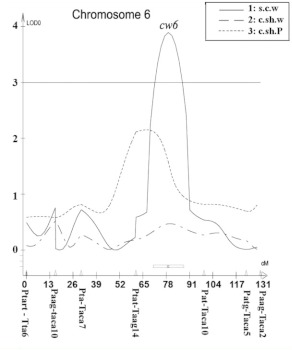
Composite interval mapping of the cocoon related traits in *Bombyx mori.* Significance thresholds are indicated by horizontal lines, with LOD = 3.0 (genome wide *P* < 0.001) as determined by 1000 permutations of the mapping data (Churchill and Doerge 1994). High quality figures are available online.

**Figure 4.**
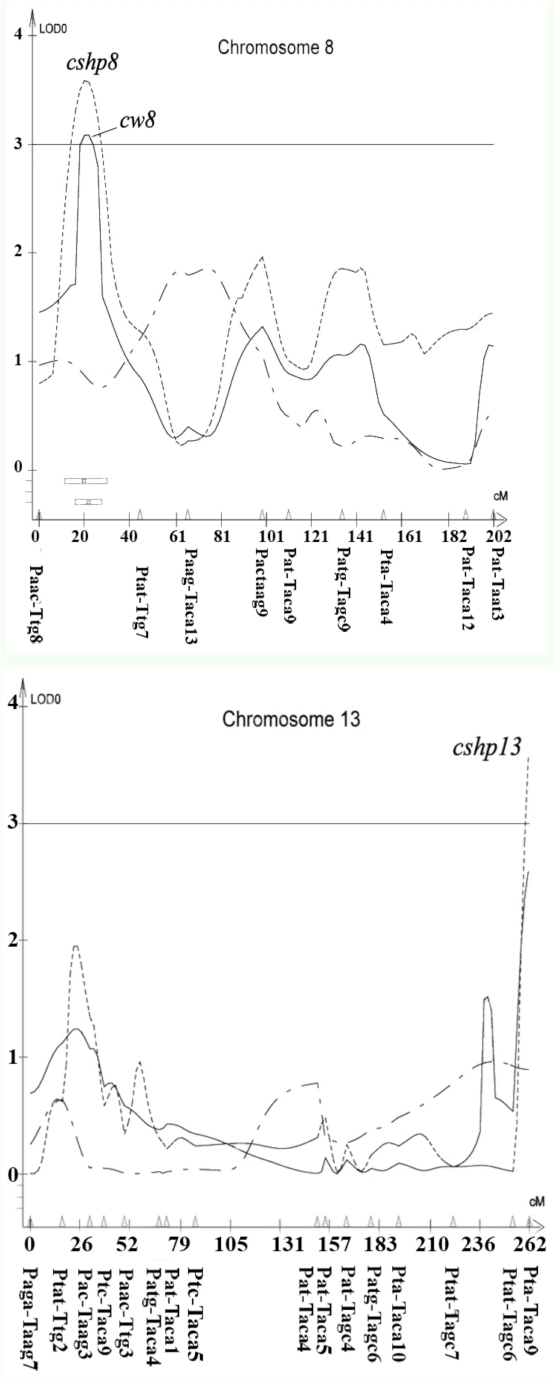

**Figure 4.**
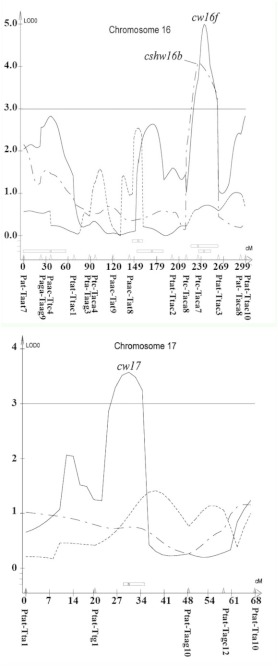

**Figure 4.**
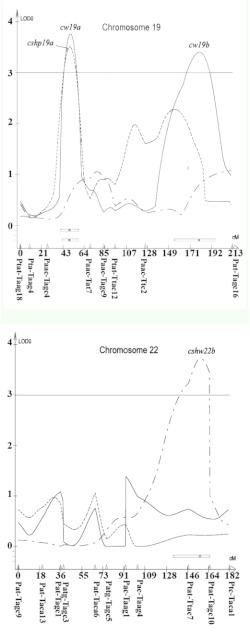

**Figure 4.**
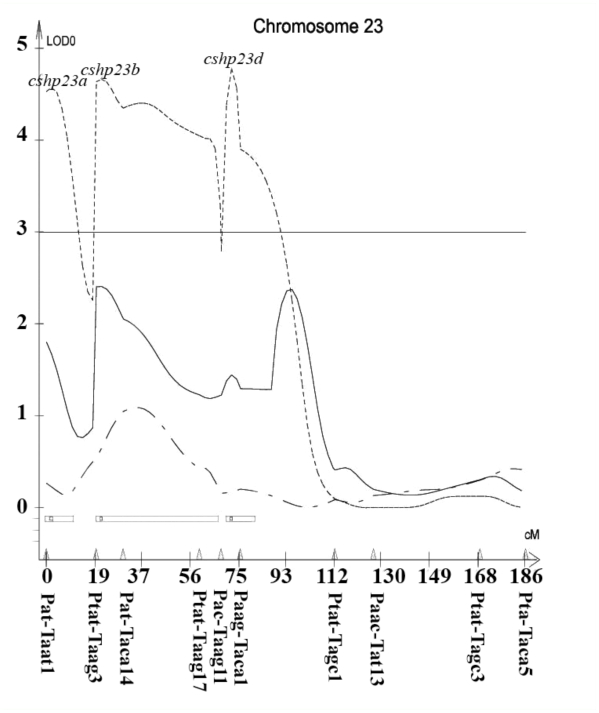

It seems that some QTL had pliotropic effects on the traits. One QTL controlling both cocoon weight and cocoon shell weight traits located on the LG16 between neighboring markers Ptc/Taca7-Ptat/Ttac3 was observed. In addition, three QTL were observed controlling both cocoon weight and cocoon shell percentage traits located on the LG8, LG16, and LG19 between neighboring markers Paac/Ttg8-Ptat/Ttg7, Paac/Tat8-Ptat/Ttac2 and Paac/Tagc4-Paac/Tat7.

A total of 19.35% of clearly readable and qualified AFLP bands were polymorphic between the P107 inbred line and the Khorasan Lemon native strain of *B. mori.* A higher level (61%) of polymorphic AFLP marker has been reported by Tan et al. (2001) in a single backcross (no. 782 and od100) family of *B. mori.* To explain this approach, they discussed several factors:

1. Employing two distinct *B. mori* strains in the present study, P107 and Khorasan Lemon are two examples of distinct silkworm inbred lines and strains. The former is from the Japanese bivoltine system and the latter is from the Iranian native monovoltine system.
2. Detecting high levels of polymorphisms by the AFLP technique (Huys et al. 1996; Latorra et al. 1996; Mackill et al. 1996; Wan et al. 1999)
3. A large fraction of the silkworm genome consists of families of transposable elements such as *Bm1, BMC1* (a member of the *LINE1* family), *mariner, mariner*-like elements (*Bmmar1*), long terminal repeat transposons (LTRs), non-long terminal transposons (nonLTRs), and others (Ueda et al. 1986; Herrer and Wang 1991; Xiong and Eickbush 1993; Xiong et al. 1993; Robertson and Asplund 1996; Tomita et al. 1997; Shimizu et al. 2000; Wang et al. 2000).

Among the 20 pairs of AFLP primer combinations applied in this study, an average of 9.2 polymorphic AFLP markers per primer combination for linkage analysis and QTL mapping was recognized. This rate was considerably lower than two other AFLP linkage and QTL mapping studies on silkworm with 35.7 (Tan et al. 2001) and 36.4 (Lu et al. 2004) fragments per primer. This may be due to the degree of differences between parental lines and strains and the primer combinations used.

The present AFLP map consisted of 24 linkage groups, whereas the haploid genome of *B. mori* has 28 chromosomes. As Promboon et al. (1995), Young et al. (1998), and He (1998) reported, this may be due to nonequivalence between the number of linkage groups and the number of chromosomes. In the RFLP based linkage map by Goldsmith (1991), 15 linkage groups were reported. However, by using morphological (Doira et al. 1992), RAPD (Yasukochi et al. 1998), RFLP (Nguu et al. 2005), AFLP (Sima et al. 2006), and SNP (Yamamoto et al. 2006) markers, 28 linkage groups and using SSR (Miao et al. 2005) markers, 29 linkage groups have been recognized in *B. mori.* It has also been shown that the large number of chromosomes in the haploid *B. mori* genome (*n* = 28), typical of Lepidoptera, makes it difficult to construct maps without missing some chromosomes (Yasukochi 1998).

The whole cocoon weight, cocoon shell weight, and cocoon shell percentage are the major economic traits in *B. mori* that are controlled by a polygene (Li et al. 2006). In the present study, a single F_2_ population derived from a cross between P107 Japanese inbred line and Khorasan Lemon Iranian native strain was used as the mapping population. Among the 21 QTL for the traits studied, one QTL had dominance effect, 13 QTL had under dominance or recessive effects, and seven QTL had over dominance effects (Table 4). Li et al. (2006) in a backcrossed population (BC1) derived from a cross between C100 and Dazao detected 40 QTL for whole cocoon weight and related traits, of which 19 were additive effect QTL and 21 were reduced effects QTL.

In summary, 159 AFLP markers were employed to construct a linkage map for *B. mori* with an average marker resolution of 19.31 cM. We identified 21 QTL (*n* = 21) using the composite interval mapping method that affects whole cocoon weight and related traits. The effects of these QTL were under dominance, dominance, and over dominance. Since AFLP amplification is highly reproducible, the development of an AFLP linkage map and subsequently the identification of strain-specific markers for tracking allele frequency changes and quantitative trait loci (QTL) analysis for economically important traits provides an invaluable tool for improving *B. mori* breeds, strains, and hybrids in order to enhance the silk production.

## Abbreviations

ABRII: Agricultural Biotechnology Research Institute of Iran;
AFLP: Amplified Fragment Length Polymorphism;
AREO: Agricultural Research and Education Organization;
BC: Backcross;
CIM: Composite Interval Mapping;
cM: centimorgans;
ESTs: Expressed Sequence Tags;
IM: Interval Mapping;
ISRC: Iran Silkworm Research Center;
LG: Linkage Group;
LOD: Logarithm of Odds;
LRT: Likelihood Ratio Test;
NPV: Nuclear Polyhidrosis Virus;
PCR: Polymerase Chain Reaction;
QTL: Quantitative Trait Loci;
RAPD: Random Amplified Polymorphic DNA;
RFLP: Restriction Fragment Length Polymorphism;
SADF: Selective Amplification of DNA Fragments;
SNP: Single Nucleotide Polymorphism
